# The *cyclo*-tetra­phosphate Cd_2_P_4_O_12_, a member of the isotypic series *M*_2_P_4_O_12_ (*M* = Mg, Mn, Fe, Co, Ni, Cu)

**DOI:** 10.1107/S1600536810040195

**Published:** 2010-10-13

**Authors:** Matthias Weil

**Affiliations:** a Institute for Chemical Technologies and Analytics, Division of Structural Chemistry, Vienna University of Technology, Getreidemarkt 9/164-SC, A-1060 Vienna, Austria

## Abstract

The title compound, Cd_2_P_4_O_12_, dicadmium *cyclo*-tetra­phosphate, crystallizes isotypically with the members of the series *M* ^II^_2_P_4_O_12_, where *M* = Mg, Mn, Fe, Co, Ni or Cu. Two CdO_6_ octa­hedra, one with 2 and one with 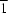 symmetry, share corners with the centrosymmetric P_4_O_12_^4−^ ring anion that is built up from four corner-sharing PO_4_ tetra­hedra. The isolated ring anions are arranged in layers parallel to (10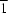) with the CdO_6_ octa­hedra situated between these layers. The main difference between the individual *M* ^II^_2_P_4_O_12_ structures pertains to the different sizes of the *M*O_6_ octa­hedra whereas the geometric parameters of all *cyclo*-P_4_O_12_^4−^ anions are very similar.

## Related literature

For a previous powder X-ray study of Cd_2_P_4_O_12_, see: Laügt *et al.* (1973 ▶). The structure of the low-temperature *α*-modification of the *catena*-polyphosphate Cd(PO_3_)_2_ was refined by Bagieu-Beucher *et al.* (1974 ▶). For isotypic *M* ^II^_2_P_4_O_12_ structures, see: Nord & Lindberg (1975 ▶) for *M* = Mg; Glaum *et al.* (2002 ▶) for Mn; Nord *et al.* (1990 ▶) and Genkina *et al.* (1985 ▶) for Fe; Nord (1982 ▶) and Olbertz *et al.* (1998 ▶) for Co; Nord (1983 ▶) and Olbertz *et al.* (1998 ▶) for Ni; Laügt *et al.* (1972 ▶) for Cu. A review on the crystal chemistry of phosphates was published by Durif (1995 ▶). Ionic radii were compiled by Shannon (1976 ▶).

## Experimental

### 

#### Crystal data

Cd_2_P_4_O_12_
*M* *_r_* = 540.68
Monoclinic, 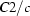

*a* = 12.3342 (2) Å
*b* = 8.6373 (2) Å
*c* = 10.4037 (2) Å
β = 119.402 (1)°
*V* = 965.59 (3) Å^3^
*Z* = 4
Mo *K*α radiation
μ = 5.13 mm^−1^
*T* = 296 K
0.36 × 0.24 × 0.12 mm

#### Data collection

Bruker APEXII CCD diffractometer
Absorption correction: multi-scan (*SADABS*; Bruker, 2008 ▶) *T*_min_ = 0.260, *T*_max_ = 0.578
11480 measured reflections
3001 independent reflections
2936 reflections with *I* > 2σ(*I*)
*R*_int_ = 0.036

#### Refinement

*R*[*F* ^2^ > 2σ(*F* ^2^)] = 0.023
*wR*(*F* ^2^) = 0.054
*S* = 1.23
3001 reflections
85 parameters
Δρ_max_ = 2.08 e Å^−3^
Δρ_min_ = −1.04 e Å^−3^

### 

Data collection: *APEX2* (Bruker, 2008 ▶); cell refinement: *SAINT* (Bruker, 2008 ▶); data reduction: *SAINT*; program(s) used to solve structure: *SHELXS97* (Sheldrick, 2008 ▶); program(s) used to refine structure: *SHELXL97* (Sheldrick, 2008 ▶); molecular graphics: *ATOMS for Windows* (Dowty, 2006 ▶); software used to prepare material for publication: *SHELXL97*.

## Supplementary Material

Crystal structure: contains datablocks I, global. DOI: 10.1107/S1600536810040195/mg2104sup1.cif

Structure factors: contains datablocks I. DOI: 10.1107/S1600536810040195/mg2104Isup2.hkl

Additional supplementary materials:  crystallographic information; 3D view; checkCIF report

## Figures and Tables

**Table 1:** Selected bond lengths (Å)

Cd1—O1^i^	2.1875 (12)
Cd1—O6	2.3034 (10)
Cd1—O2^ii^	2.3690 (11)
Cd2—O5^iii^	2.2037 (12)
Cd2—O6	2.2563 (11)
Cd2—O2	2.2809 (11)
P1—O1	1.4624 (12)
P1—O2	1.5052 (11)
P1—O3^iv^	1.5840 (12)
P1—O4	1.5983 (11)
P2—O5	1.4604 (12)
P2—O6	1.5011 (11)
P2—O3	1.5848 (12)
P2—O4	1.5918 (12)

Symmetry codes: (i) 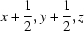; (ii) 
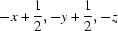
; (iii) 
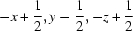
; (iv) 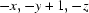.

**Fig. 1.**
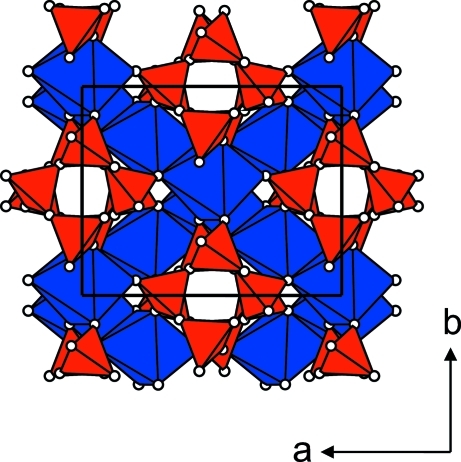
The crystal structure of Cd2P4O12 in a projection along [001]. PO4 tetrahedra are red, CdO6 octahedra are blue and O atoms are displayed as white spheres.

**Fig. 2.**
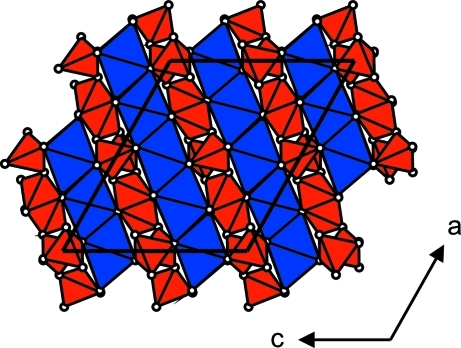
The crystal structure of Cd2P4O12 in a projection along [010]. Colour code as in Fig. 1.

**Fig. 3.**
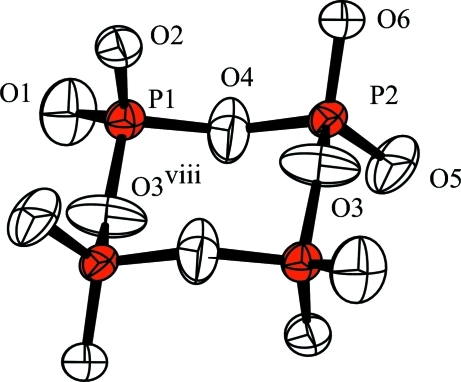
The P4O12 ring anion with displacement ellipsoids drawn at the 99% level. Non-labelled atoms are generated by inversion symmetry. [Symmetry code: (viii) -x, -y + 1, -z.]

## supplementary crystallographic information

### Comment

*M*^II^_2_P_4_O_12_ compounds containing the *cyclo*-tetraphosphate anion P_4_O_12_^4-^ have been the subject of numerous crystallographic studies. Except for Cd_2_P_4_O_12_ (X-ray powder data; Laügt *et al.*, 1973), detailed structure data are available for Mg_2_P_4_O_12_ (Nord & Lindberg, 1975), Mn_2_P_4_O_12_ (Glaum *et al.*, 2002), Fe_2_P_4_O_12_ (Nord *et al.*, 1990; Genkina *et al.*, 1985), Co_2_P_4_O_12_ (Nord, 1982; Olbertz *et al.*, 1998), Ni_2_P_4_O_12_ (Nord, 1983; Olbertz *et al.*, 1998) and Cu_2_P_4_O_12_ (Laügt *et al.*, 1972). During experiments intended for crystal growth of large single crystals of the low-temperature modification of cadmium *catena*-polyphosphate, *α*-Cd(PO_3_)_2_ (Bagieu-Beucher *et al.*, 1974), single crystals of the title compound were obtained instead.

The crystal structures of the isotypic *M*^II^_2_P_4_O_12_ family are built up from centrosymmetric P_4_O_12_^4-^ ring anions. The isolated anions are arranged in layers parallel to (101). Two sets of slightly distorted *M*O_6_ octahedra, one with 1 symmetry and one with 2 symmetry, share edges and are situated in the interlayer space. The three-dimensional framework is accomplished by corner-sharing of the *M*O_6_ units and the P_4_O_12_^4-^ anions. Figures 1 and 2 show the resulting arrangement for Cd_2_P_4_O_12_.

The P_4_O_12_^4-^ ring anion of Cd_2_P_4_O_12_ (Fig. 3) consists of four corner-sharing PO_4_ tetrahedra and shows the typical features with respect to bond lengths and angles, *i.e.* shorter terminal P—O bonds and longer P—O bonds to the bridging O atoms. A review on structures containing the *cyclo*-tetraphosphate anion has been given by Durif (1995) where characteristic distances and angles are compiled. The individual bond lengths and angles of the P_4_O_12_^4-^ anions are very similar in all *M*^II^_2_P_4_O_12_ structures. The main difference between the structures is related to the varying ionic radii of the *M*^II^ cations. Correspondingly, the *M*O_6_ octahedra show (slight) variations in the *M*—O bond lengths. In the *M*^II^_2_P_4_O_12_ family (*M* = Mg, Mn, Fe, Co, Ni, Cu, and Cd), Cd^II^ has the largest ionic radius (0.95 Å) for coordination number 6 (Shannon, 1976). This value seems to be the upper limit for the existence of the *M*^II^_2_P_4_O_12_ family of structures. For larger *M*^II^ cations like Hg^II^ or Pb^II^ (ionic radius 1.02 Å and 1.19 Å, respectively) solely long-chain *catena*-polyphosphate structures *M*(PO_3_)_2_ are realised.

In the review on condensed phosphates given by Durif it was stated that *cyclo*-Cd_2_P_4_O_12_ transforms irreversibly into the low-temperature *α*-modification of the long-chain polyphosphate Cd(PO_3_)_2_ by prolonged heating at 573 K (Durif, 1995, and references therein), indicating that this transformation process is kinetically controlled. This assumption is confirmed by DSC (differential scanning calorimetry) measurements of the current sample (N_2_ atmosphere, heating rate 10 K^.^min^-1^). Whereas no phase transition has been observed for this compound up to 873 K under these conditions, heating the sample at 873 K in a laboratory furnace under atmospheric conditions for 20 h resulted in a complete transformation into *α*-Cd(PO_3_)_2_.

### Experimental

Single crystals suitable for X-ray structure analysis were grown using the phosphate flux method. CdO (0.7 g) was placed in a glassy carbon crucible and was covered carefully with 70%_wt_ H_3_PO_4_ (5.4 g). The crucible was subjected to the following temperature programme: RT → 693 K [3 h] → 693 K [5 h] → 573 K [48 h]. Then the crucible was removed from the furnace. Prismatic colourless crystals with maximum edge lengths of 1.5 mm were obtained by leaching the phosphate flux with warm water.

### Refinement

The highest peak in the final Fourier map is located 0.62 Å from Cd2 and the deepest hole is 0.96 Å from the same atom.

### Crystal data

**Table:**

Cd_2_P_4_O12	*F*(000) = 1008
*M**_r_* = 540.68	*D*_x_ = 3.719 Mg m^−^^3^
Monoclinic, *C*2/*c*	Mo *K*α radiation, λ = 0.71073 Å
Hall symbol: -C 2yc	Cell parameters from 9733 reflections
*a* = 12.3342 (2) Å	θ = 3.0–40.1°
*b* = 8.6373 (2) Å	µ = 5.13 mm^−^^1^
*c* = 10.4037 (2) Å	*T* = 296 K
β = 119.402 (1)°	Fragment, colourless
*V* = 965.59 (3) Å^3^	0.36 × 0.24 × 0.12 mm
*Z* = 4	

### Data collection

**Table:**

Bruker APEXII CCD diffractometer	3001 independent reflections
Radiation source: fine-focus sealed tube	2936 reflections with *I* > 2σ(*I*)
graphite	*R*_int_ = 0.036
ω and φ scans	θ_max_ = 40.1°, θ_min_ = 3.0°
Absorption correction: multi-scan (*SADABS*; Bruker, 2008)	*h* = −22→22
*T*_min_ = 0.260, *T*_max_ = 0.578	*k* = −13→15
11480 measured reflections	*l* = −18→18

### Refinement

**Table:**

Refinement on *F*^2^	Primary atom site location: structure-invariant direct methods
Least-squares matrix: full	Secondary atom site location: difference Fourier map
*R*[*F*^2^ > 2σ(*F*^2^)] = 0.023	*w* = 1/[σ^2^(*F*_o_^2^) + (0.0182*P*)^2^ + 0.873*P*] where *P* = (*F*_o_^2^ + 2*F*_c_^2^)/3
*wR*(*F*^2^) = 0.054	(Δ/σ)_max_ = 0.001
*S* = 1.23	Δρ_max_ = 2.08 e Å^−^^3^
3001 reflections	Δρ_min_ = −1.04 e Å^−^^3^
85 parameters	Extinction correction: *SHELXL97* (Sheldrick, 2008), Fc^*^=kFc[1+0.001xFc^2^λ^3^/sin(2θ)]^-1/4^
0 restraints	Extinction coefficient: 0.0306 (6)

### Special details

**Table:**

Geometry. All e.s.d.'s (except the e.s.d. in the dihedral angle between two l.s. planes) are estimated using the full covariance matrix. The cell e.s.d.'s are taken into account individually in the estimation of e.s.d.'s in distances, angles and torsion angles; correlations between e.s.d.'s in cell parameters are only used when they are defined by crystal symmetry. An approximate (isotropic) treatment of cell e.s.d.'s is used for estimating e.s.d.'s involving l.s. planes.
Refinement. Refinement of *F*^2^ against ALL reflections. The weighted *R*-factor *wR* and goodness of fit *S* are based on *F*^2^, conventional *R*-factors *R* are based on *F*, with *F* set to zero for negative *F*^2^. The threshold expression of *F*^2^ > σ(*F*^2^) is used only for calculating *R*-factors(gt) *etc*. and is not relevant to the choice of reflections for refinement. *R*-factors based on *F*^2^ are statistically about twice as large as those based on *F*, and *R*- factors based on ALL data will be even larger.

### Fractional atomic coordinates and isotropic or equivalent isotropic displacement parameters (Å^2^)

**Table:**

	*x*	*y*	*z*	*U*_iso_*/*U*_eq_	
Cd1	0.5000	0.463931 (16)	0.2500	0.01089 (4)	
Cd2	0.2500	0.2500	0.0000	0.00995 (4)	
P1	−0.01063 (3)	0.27016 (4)	0.02086 (4)	0.00915 (6)	
P2	0.18879 (3)	0.50069 (4)	0.19159 (3)	0.00957 (6)	
O1	−0.04856 (12)	0.13811 (15)	0.07757 (14)	0.0192 (2)	
O2	0.04150 (11)	0.24122 (13)	−0.08092 (13)	0.01274 (17)	
O3	0.12504 (11)	0.61475 (16)	0.05498 (13)	0.0208 (2)	
O4	0.08602 (11)	0.37174 (16)	0.15789 (13)	0.0188 (2)	
O5	0.21814 (13)	0.57449 (17)	0.33127 (13)	0.0203 (2)	
O6	0.29305 (9)	0.42682 (14)	0.17838 (12)	0.01323 (16)	

### Atomic displacement parameters (Å^2^)

**Table:**

	*U*^11^	*U*^22^	*U*^33^	*U*^12^	*U*^13^	*U*^23^
Cd1	0.01110 (6)	0.00998 (7)	0.01157 (6)	0.000	0.00555 (4)	0.000
Cd2	0.01135 (6)	0.01049 (7)	0.00858 (6)	0.00011 (3)	0.00534 (4)	0.00030 (3)
P1	0.01011 (12)	0.00779 (13)	0.01007 (12)	0.00002 (9)	0.00536 (9)	0.00080 (9)
P2	0.00955 (12)	0.00927 (14)	0.00907 (11)	0.00068 (10)	0.00393 (9)	−0.00214 (9)
O1	0.0242 (5)	0.0138 (5)	0.0198 (4)	−0.0032 (4)	0.0111 (4)	0.0051 (4)
O2	0.0118 (4)	0.0150 (5)	0.0129 (4)	−0.0001 (3)	0.0072 (3)	−0.0029 (3)
O3	0.0209 (5)	0.0247 (6)	0.0205 (5)	0.0139 (4)	0.0132 (4)	0.0109 (4)
O4	0.0198 (4)	0.0225 (5)	0.0176 (4)	−0.0106 (4)	0.0120 (4)	−0.0095 (4)
O5	0.0245 (5)	0.0202 (6)	0.0146 (4)	−0.0011 (4)	0.0084 (4)	−0.0098 (4)
O6	0.0102 (3)	0.0129 (4)	0.0156 (4)	0.0016 (3)	0.0056 (3)	−0.0030 (3)

### Geometric parameters (Å, °)

**Table:**

Cd1—O1^i^	2.1875 (12)	Cd2—Cd1^iv^	3.4370
Cd1—O1^ii^	2.1875 (12)	P1—O1	1.4624 (12)
Cd1—O6^iii^	2.3034 (10)	P1—O2	1.5052 (11)
Cd1—O6	2.3034 (10)	P1—O3^viii^	1.5840 (12)
Cd1—O2^iv^	2.3690 (11)	P1—O4	1.5983 (11)
Cd1—O2^v^	2.3690 (11)	P2—O5	1.4604 (12)
Cd1—Cd2	3.4370	P2—O6	1.5011 (11)
Cd1—Cd2^iii^	3.4370	P2—O3	1.5848 (12)
Cd2—O5^vi^	2.2037 (12)	P2—O4	1.5918 (12)
Cd2—O5^vii^	2.2037 (12)	O1—Cd1^ix^	2.1875 (12)
Cd2—O6^iv^	2.2563 (11)	O2—Cd1^iv^	2.3690 (11)
Cd2—O6	2.2563 (11)	O3—P1^viii^	1.5840 (12)
Cd2—O2^iv^	2.2809 (11)	O5—Cd2^i^	2.2037 (12)
Cd2—O2	2.2809 (11)		
			
O1^i^—Cd1—O1^ii^	93.10 (7)	O5^vii^—Cd2—O2	90.06 (5)
O1^i^—Cd1—O6^iii^	90.95 (4)	O6^iv^—Cd2—O2	84.63 (4)
O1^ii^—Cd1—O6^iii^	100.06 (4)	O6—Cd2—O2	95.37 (4)
O1^i^—Cd1—O6	100.06 (4)	O2^iv^—Cd2—O2	180.00 (6)
O1^ii^—Cd1—O6	90.95 (4)	O1—P1—O2	119.08 (8)
O6^iii^—Cd1—O6	164.00 (6)	O1—P1—O3^viii^	107.89 (8)
O1^i^—Cd1—O2^iv^	174.69 (4)	O2—P1—O3^viii^	109.73 (6)
O1^ii^—Cd1—O2^iv^	91.90 (5)	O1—P1—O4	108.33 (7)
O6^iii^—Cd1—O2^iv^	86.40 (4)	O2—P1—O4	109.24 (6)
O6—Cd1—O2^iv^	81.64 (4)	O3^viii^—P1—O4	101.05 (8)
O1^i^—Cd1—O2^v^	91.90 (5)	O5—P2—O6	118.14 (7)
O1^ii^—Cd1—O2^v^	174.69 (4)	O5—P2—O3	113.12 (8)
O6^iii^—Cd1—O2^v^	81.64 (4)	O6—P2—O3	104.61 (6)
O6—Cd1—O2^v^	86.40 (4)	O5—P2—O4	107.83 (7)
O2^iv^—Cd1—O2^v^	83.17 (6)	O6—P2—O4	107.92 (7)
O5^vi^—Cd2—O5^vii^	180.0	O3—P2—O4	104.28 (8)
O5^vi^—Cd2—O6^iv^	93.87 (5)	P1—O1—Cd1^ix^	148.67 (8)
O5^vii^—Cd2—O6^iv^	86.13 (5)	P1—O2—Cd2	121.90 (7)
O5^vi^—Cd2—O6	86.13 (5)	P1—O2—Cd1^iv^	129.45 (7)
O5^vii^—Cd2—O6	93.87 (5)	Cd2—O2—Cd1^iv^	95.30 (4)
O6^iv^—Cd2—O6	180.00 (5)	P1^viii^—O3—P2	139.93 (8)
O5^vi^—Cd2—O2^iv^	90.06 (5)	P2—O4—P1	138.17 (8)
O5^vii^—Cd2—O2^iv^	89.94 (5)	P2—O5—Cd2^i^	162.37 (10)
O6^iv^—Cd2—O2^iv^	95.37 (4)	P2—O6—Cd2	119.78 (6)
O6—Cd2—O2^iv^	84.63 (4)	P2—O6—Cd1	140.75 (7)
O5^vi^—Cd2—O2	89.94 (5)	Cd2—O6—Cd1	97.83 (4)

Symmetry codes: (i) −*x*+1/2, *y*+1/2, −*z*+1/2; (ii) *x*+1/2, *y*+1/2, *z*; (iii) −*x*+1, *y*, −*z*+1/2; (iv) −*x*+1/2, −*y*+1/2, −*z*; (v) *x*+1/2, −*y*+1/2, *z*+1/2; (vi) −*x*+1/2, *y*−1/2, −*z*+1/2; (vii) *x*, −*y*+1, *z*−1/2; (viii) −*x*, −*y*+1, −*z*; (ix) *x*−1/2, *y*−1/2, *z*.
